# Do Xpert MTB/RIF Cycle Threshold Values Provide Information about Patient Delays for Tuberculosis Diagnosis?

**DOI:** 10.1371/journal.pone.0162833

**Published:** 2016-09-09

**Authors:** Willy Ssengooba, Durval Respeito, Edson Mambuque, Silvia Blanco, Helder Bulo, Inacio Mandomando, Bouke C. de Jong, Frank G. Cobelens, Alberto L. García-Basteiro

**Affiliations:** 1 Department of Global Health and Amsterdam Institute of Global Health and Development, Academic Medical Center, University of Amsterdam, Amsterdam, the Netherlands; 2 ISGlobal, Barcelona Ctr. Int. Health Res. (CRESIB), Hospital Clínic—Universitat de Barcelona, Barcelona, Spain; 3 Department of Medical Microbiology, College of Health Sciences Makerere University, Kampala, Uganda; 4 Centro de Investigação em Saúde de Manhiça, Maputo, Mozambique; 5 Instituto Nacional de Saúde, Ministério da Saúde, Maputo, Mozambique; 6 Mycobacteriology Unit, Institute of Tropical Medicine, Antwerp, Belgium; 7 KNCV Tuberculosis Foundation, The Hague, the Netherlands; Universidad Nacional de la Plata, ARGENTINA

## Abstract

**Introduction:**

Early diagnosis and initiation to appropriate treatment is vital for tuberculosis (TB) control. The XpertMTB/RIF (Xpert) assay offers rapid TB diagnosis and quantitative estimation of bacterial burden through Cycle threshold (Ct) values. We assessed whether the Xpert Ct value is associated with delayed TB diagnosis as a potential monitoring tool for TB control programme performance.

**Materials and Methods:**

This analysis was nested in a prospective study under the routine TB surveillance procedures of the National TB Control Program in Manhiça district, Maputo province, Mozambique. Presumptive TB patients were tested using smear microscopy and Xpert. We explored the association between Xpert Ct values and self-reported delay of Xpert-positive TB patients as recorded at the time of diagnosis enrolment. Patients with >60 days of TB symptoms were considered to have long delays.

**Results:**

Of 1,483 presumptive TB cases, 580 were diagnosed as TB of whom 505 (87.0%) were due to pulmonary TB and 302 (94.1%) were Xpert positive. Ct values (range, 9.7–46.4) showed a multimodal distribution. The median (IQR) delay was 30 (30–45) days. Ct values showed no correlation with delay (R^2^ = 0.001, p = 0.621), nor any association with long delays: adjusted odds ratios (AOR) (95% confidence interval [CI]) comparing to >28 cycles 0.99 (0.50–1.96; p = 0.987) for 23–28 cycles, 0.93 (0.50–1.74; p = 0.828) for 16–22 cycles; and 1.05 (0.47–2.36; p = 0.897) for <16 cycles. Being HIV-negative (AOR [95% CI]), 2.05 (1.19–3.51, p = 0.009) and rural residence 1.74 (1.08–2.81, p = 0.023), were independent predictors of long delays.

**Conclusion:**

Xpert Ct values were not associated with patient delay for TB diagnosis and cannot be used as an indicator of TB control program performance.

## Introduction

Tuberculosis (TB) remains a global health burden and caused 1.5 million deaths in 2014 globally [1]. Early diagnosis and initiation of effective TB treatment are vital for bringing down TB transmission [2,3]. Delays in diagnosing TB hamper effective TB control, and TB control programmes aim for reducing such delays as much as possible [4]. A readily available measure of diagnostic delay would help TB control programmes in monitoring their performance in this respect.

The Xpert MTB/RIF assay (Xpert), a cartridge-based real-time PCR, offers rapid diagnosis of susceptible TB and resistance-conferring mutations to rifampicin in the *rpoB* gene within 2 hours of testing. The Xpert technology also offers a quantitative estimation of bacterial burden in form of cycle threshold (Ct) values. Ct values have been found to predict decreasing bacterial burden with increasing immunosuppression and a combination of Ct values and sputum smear status to be a possible measure of bacterial burden in high HIV-burden settings [5]. Several studies have documented the various factors associated with patient delay for TB diagnosis [6,7,8,9,10,11,12,13,14]. Mycobacterial load is likely to increase with patient delay since bacillary burden has been found to grow over time and may have an impact on the novel diagnostic tests [15,16].

Mozambique with a population of 27 million people in 2014, has low case detection rate, below 50%[17], the estimated TB incidence is around 551 cases per 100,000 population; of these cases, an estimated 58% are HIV co-infected [18]. By 2014, 24 Xpert machines were operating in the country [1]. A study done in Beira city of Mozambique found the median patient delay for TB diagnosis to be 61 days (28–113)[19].

This study aimed at analyzing whether Ct values are correlated with diagnostic delays and/or associated with long delays among patients referred for Xpert testing in Mozambique. If such correlation or association were to exist, Ct values could be used as an indicator for monitoring diagnostic delays.

## Materials and Methods

### Study setting

The current study was nested in a prospective surveillance based study embedded in the routine TB patient management procedures of the National TB Control Program for the district of Manhiça, a high TB and HIV burden area in Southern Mozambique [20,21]. The study was conducted by the Manhiça Health Research Center (Centro de Investigação em Saude de Manhiça, CISM) located adjacent to the Manhiça District Hospital, where a majority of the presumptive TB cases from peripheral health centres in the district are referred for diagnosis.

### Study participants

The study enrolled consecutively presumptive TB cases referred for TB diagnosis at the Manhiça District Hospital from 11 health care centers between August 2013 and August 2014. A presumptive TB case was defined as someone presenting with cough for more than 2 weeks and/or weight loss and/or fever and/or night sweats for more than two weeks, regardless of having been previously treated for TB. Although Xpert was not routinely used in the country at that time, it was used in this research context, together with AFB smear on Ziehl Neelsen microscopy, as an initial test for any specimen (mostly sputa) provided by a presumptive TB case. Culture was only done for diagnosis in TB previously treated patients (former WHO Category 2 patients) or new TB cases who were AFB smear positive at month 5 of follow up. AFB smears were processed in the Biosafety Level III TB laboratory at CISM. On a ratio of 1:2 mL of sample to sample reagents, incubated at room temperature for 15 minutes, 1 mL of the mixture was added to the Xpert cartridge and analyzed in Xpert system according to manufacturer’s guide. Xpert Ct values were taken as the mean of PCR cycles obtained from the five probes (A-E) of the Xpert machines. Ct values inversely correlate with bacterial load i.e. lower Ct values represent a higher starting concentration of DNA template whereas higher Ct values represent a lower concentration of DNA template. The mean Ct values are also categorized by the Xpert system semi-quantitatively in relation to sample positivity as very low (>28 cycles), low (23–28 cycles), medium (16–22 cycles) and high (<16 cycles)[22].

Study specific questionnaires were implemented to capture the most important variables. Demographic and clinical data were collected at enrolment.

### Operational definitions

Patient delay for TB diagnosis was defined as reported total duration of symptoms at the time of study enrolment. By the time of enrolment the patient could have had several diagnostic visits to the clinic where the current TB diagnosis was made, as well as sought care from several health services providers. For these reasons as well as the wide variation in literature about patient delay for TB diagnosis [6,7,8,9,10,11,12,13,14], we operationalized long delay to be ≥ 60 days with TB symptoms before TB diagnosis before TB diagnosis before current study data analysis. This was considered as delay long enough to significantly impact on the TB control programmes.

We considered cough as the main symptom and if absent, duration of the other TB symptom(s) was considered. For the current analysis, a TB case was defined as a study participant positive with Xpert test.

### Data management and analysis

The data collected from presumptive TB cases and TB cases were kept in paper forms (CRFs), which were checked by the study coordinators and study nurse at the end of each week for quality assurance. Completed forms were double computer-entered at CISM. Data were exported to Stata, (Stata Corp LP, College Station TX, USA) for analysis of frequencies and proportions of participants’ characteristics and median symptom duration were compared. We used the Wilcoxon ranksum test and the Kruskall-Wallis test to compare delays between groups, and Spearman’s correlation coefficient to express the correlation between delays and Ct values. In the analysis of long delays we adjusted the association between categorized Ct values and delays of ≥60 days duration for confounding variables in a multivariate logistic regression model. In a secondary analysis we explored independent associations with long delays by including in a similar model all covariates with a p-value <0.2 at univariate analysis, using back-ward elimination to arrive at a final model that contained variables with a p-value <0.05. Reported model p-values were based on the likelihood ratio test.

### Ethical consideration

The study was approved by the CISM’s Internal Scientific Committee, CISM’s Institutional Bioethics Committee for Health (CIBS—Comité Institucional de Bioética para a Saúde) and the National Bioethics Committee for Health (CNB—Comité Nacional de Bioética para a Saúde) of Mozambique. All individuals gave a written informed consent before participation in the study.

## Results

### Characteristics of participants

**Fig 1:**
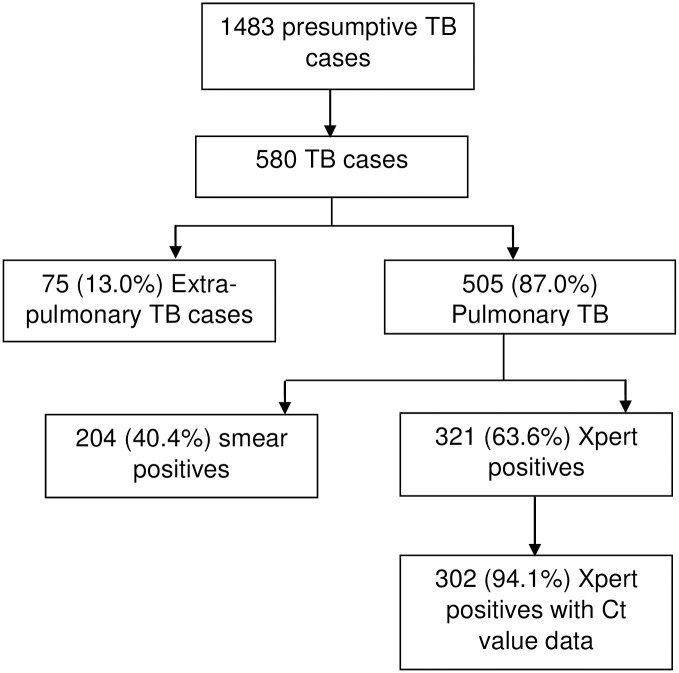
Flow diagram showing XpertMTB/RIF test positive tuberculosis participants. TB = tuberculosis, EPTB = extra pulmonary tuberculosis, PTB = pulmonary tuberculosis.

A total of 1483 presumptive TB cases were enrolled in the current study of whom 580 were diagnosed as TB. 505(87.0%) were due to pulmonary TB, 204 (40.4%) of whom were smear positive and 321 (63.6%) were positive for Xpert, Fig 1.

Of the 321 Xpert positive TB patients recorded, data for Ct value were available for 302 TB patients of whom 176 (58%) were male, median age was 35 (interquartile range 33–37) years, 154 (51%) were from urban areas of the district 222 (74%) were HIV-positive and 14 (4.6%) had *rpoB* mutation. The patient characteristics and median delay are shown in Table 1.

**Table 1:** Demographic and clinical-demographic characteristics and diagnostic delay of pulmonary TB patients.

Characteristic	Frequency, n (%)	Diagnostic delay Median (IQR)	P value
**Sex**			
Female	126(41.7)	30 (30–60)	
Male	176 (58.3)	30 (30–60)	0.555
**Age category**			
≤35	146 (48.5)	30 (30–60)	
>35	155 (51.5)	30 (30–45)	0.934
Median age (IQR)	35 (33–37)		
**Residence**			
Urban	154 (51.0)	30 (30–30)	
Rural	148 (49.0)	60 (30–60)	0.041
**History of TB treatment**			
New	254 (84.1)	30 (30–45)	
Previously treated	48 (15.9)	45(30–60)	0.177
**Presence of cough**			
Yes	290 (96.0)	30 (7–131)	
No	12 (4.0)	30 (30–53)	0.834
**Fever**			
Yes	248 (82.1)	30 (30–60)	0.854
No	54 (17.9)	30 (30–60)	
**Night sweats**			
Yes	246 (81.7)	30 (30–60)	
No	55 (18.3)	30 (30–60)	0.790
**HIV-status**			
Positive	222 (74.5)	30 (30–60)	
Negative	76 (25.5)	60 (30–60)	0.093
**ON ART**			
Yes	80 (36.0)	30 (30–60)	0.160
No	142 (64.0)	30 (30–30)	
**CD4 cell count**			
>200 cells/mm^3^	84 (44.9)	30 (30–45)	
100–200 cells/mm^3^	35 (18.7)	30 (30–60)	0.708
<100 cells/mm^3^	68 (36.4)	30 (30–41)	0.729
**Taking cotrimoxazole**			
Yes	28 (14.1)	30 (21–60)	
No	170 (85.9)	30 (30–30)	0.825
**Smear results**			
Negative	119 (39.7)	30 (30–30)	0.086
Positive	181 (60.3)	30 (30–60)	
**XpertMTB/RIF Ct value**			
Very low (>28 cycles)	73 (24.2)	30 (30–60)	
Low (23–28 cycles)	75 (24.8)	30 (30–60)	0.562
Medium (16–22 cycles)	114 (37.7)	30 (30–60)	0.991
High (<16 cycles)	40 (13.3)	30 (30–60)	0.951

TB = tuberculosis, IQR = Interquartile range. Ct = cycle threshold

### Distribution of patient delay and Ct values

**Fig 2:**
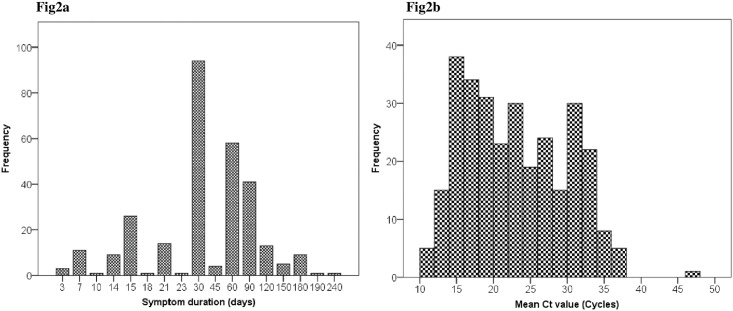
2a: Distribution of the reported symptom duration, Fig2b: Distribution of the Xpert mean Ct values.

**Fig 3:**
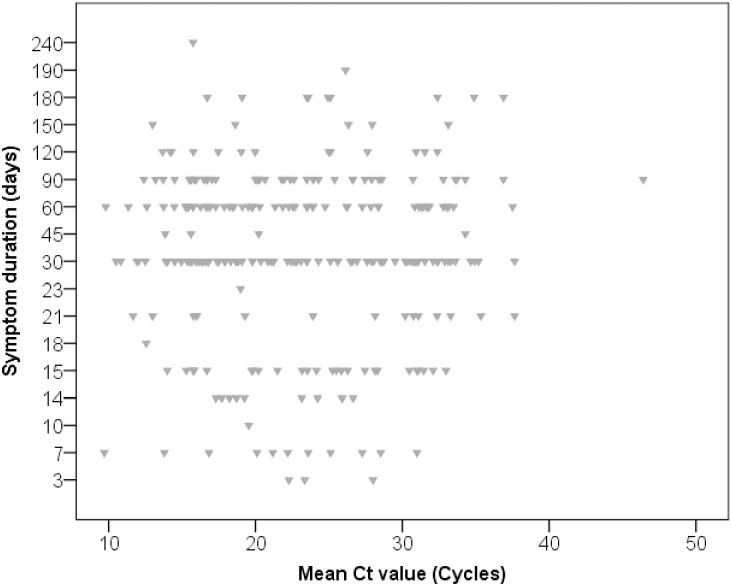
Scatter plot of individuals’ mean CT value among those testing XpertMTB/RIF positive by reported symptom duration (Spearman R^2^ = 0.001, p = 0.612).

Symptom duration showed a skewed distribution with a median of 30 days and an interquartile range of 30 to 45 days,Fig 2a; 37% had medium (16–22 cycles) Xpert Ct values. Ct values showed a multimodal distribution with peaks around values of 15, 25 and 33 cycles, Fig 2b. There was no correlation between Ct values and delays (Spearman R^2^ = 0.001, p = 0.612, Fig 3). Log-transforming the delays did not improve the correlation (R^2^ <0.001, p = 0.798).

### Association of long diagnostic delay with Ct values

In univariate analysis, Xpert Ct values showed no significant association with long delays, with adjusted odds ratios (AOR) (95% confidence interval [CI]) comparing to >28 cycles 0.99 (0.50–1.96; p = 0.987) for 23–28 cycles, 0.93 (0.50–1.74; p = 0.828) for 16–22 cycles; and 1.05 (0.47–2.36; p = 0.897) for <16 cycles. After multivariate adjustment for confounding by residence and HIV status the association remained absent, Table 2.

**Table 2:** Multivariate predictors of patient delay for TB diagnosis in Manhiça district, Maputo Mozambique.

Variables	Odds ratio	P value	[95% CI]
Ct 23–28 Cycles	0.99	0.987	0.504–1.962
Ct 16–22 Cycles	0.93	0.828	0.499–1.745
Ct <16 Cycles	1.05	0.897	0.471–2.362
HIV-negative	2.05	0.009	1.193–3.512
Rural residence	1.74	0.023	1.078–2.811

Ct = cycle threshold, CI = Confidence interval

### Predictors of long diagnostic delay

Delays (median, IQR) differed significantly between patients residing in rural parts of the district (60, 30–60) and patients residing in urban parts (30, 30–30; p = 0.041). There were no significant differences among other demographic and patient characteristics (Table 1).

Factors associated with long patient delay in the multivariate analysis also included being HIV-negative (AOR [95% CI]), 2.05 (1.19–3.51, p = 0.009) and residence in rural area of Manhiça district 1.74 (1.08–2.81, p = 0.023), Table 2.

## Discussion

We found that among pulmonary TB patients in Manhiça district of Mozambique referred for Xpert testing, Xpert Ct values were not associated with the reported duration of TB symptom duration, nor did they predict long delays for TB diagnosis of >60 days. Long delays were independently associated with rural residence and HIV-negative status.

Few studies have looked at additional information that can be generated from routine use of the Xpert assay to inform TB control programs. Previous study found Xpert Ct values to correlate with bacillary burden in the lungs, i.e. increasing bacillary load with decreasing Xpert Ct values [5]. Measures to estimate patient delay independently from what is reported by the patient would be useful as a tool for monitoring TB control program performance with regard to reducing diagnostic delays. Clearly, Xpert Ct values cannot be used for this purpose.

The lack of observed association between Ct values and diagnostic delays may imply that longer delays do not lead to higher bacterial burden, at least not at individual patient level, and that it is the individual clinical trajectory rather than duration of disease that predicts the bacterial burden at diagnosis [23,24]. Alternatively, or in addition, it may imply that the reported duration of symptoms does not reflect the duration of disease. This may be due to poor recall by patients and/or relatively long and variable periods of subclinical disease before symptoms arise[25], although subclinical disease is more likely to be missed in routine than in provider-initiated symptom screening for tuberculosis [26]. We investigated other predictors of patient delay for TB diagnosis and found rural residence and being HIV-negative to predict patient delay for TB diagnosis. It is worth noting that Xpert testing in Manhiça is only available in the district town, so rural patients first need to be referred and then take time to travel to town for the Xpert test. This was in agreement with a previous study that found rural residence as a risk factor of patient delay for TB diagnosis [13,27]. Previous studies documented HIV-positive individuals to have less patient delay compared to HIV-negative [12,28]. Previous studies have noted that HIV-positive individuals are more likely to suffer serious TB symptoms which prompt them to seek care earlier than HIV-negative individuals [26,28]. An alternative hypothesis is that TB patients who are HIV-positive and wait too long to be diagnosed die at home. Since there is capacity for smear microscopy in peripheral health centers in Manhiça district, replacing AFB with centralized Xpert may increase TB diagnostic delays [29].

Our study has some limitations. First, the delay as reported by patients was retrospectively analysed from the collected data. The reported duration may have suffered from recall bias for delays and “digit preference” (e.g. 2 weeks, 2 months) in delay ascertainment. Secondly, the findings may not represent delays among presumptive TB patients in Mozambique, but rather a selection of patients in the district referred for Xpert reflecting the local diagnostic and referral algorithm. This limits the conclusions drawn from our secondary analysis but not from our primary analysis, since the latter aimed to look at a biological phenomenon that is expected to exist independent of representativeness of the patients included in the current study. We did not collect data concerning the intervals of health care contacts to the current study yet patients may have contacted other health care facilities and received antibiotics, which may have resolved partly some TB symptoms before the current visit. Finally, we took the mean CT values from the five probes of Xpert Ct for predicting patient delay and had low prevalence 14/302 (4.6%) of *rpoB* mutation, however, in settings with high rifampicin resistance, these may be analysed separately or participants eliminated from the analysis as rifampicin resistance may alter the results.

In conclusion, in our study Xpert Ct values did not predict patient delay for TB diagnosis, and are not useful for monitoring TB control program performance with regard to reducing diagnostic delay.
